# Brand Differences in Underage Tobacco Use as Evidence for Targeted Sanctions—Reviving the Lookback

**DOI:** 10.1001/jamahealthforum.2023.3463

**Published:** 2023-10-06

**Authors:** Abigail S. Friedman, Alex C. Liber

**Affiliations:** 1 Department of Health Policy and Management, Yale School of Public Health, Yale University, New Haven, Connecticut; 2 Lombardi Comprehensive Cancer Center, Department of Oncology, Georgetown University School of Medicine, Washington, District of Columbia

## Abstract

This survey study uses data from the 2020-2021 National Survey on Drug Use and Health to estimate brand differences in underage cigarette and cigar use in the US.

## Introduction

US federal, state, and local governments rely on tobacco control policies to curtail tobacco use, particularly among youths. Yet underage combustible tobacco use remains a public health issue despite substantial declines in youth smoking and federal adoption of 21 years as the minimum legal sales age for tobacco products.^1,2,3^ This study considered the extent to which a small number of products are responsible for disproportionate shares of underage cigarette and cigar use.

## Methods

This survey study was designated non–human participants research by Yale University, and institutional review board approval was not required.

We used 2020-2021 National Survey on Drug Use and Health (NSDUH) data to estimate consumption-weighted cigarette and cigar use by age, weighted by cigarettes per month and cigar smoking days per month (NSDUH elicits frequency but not intensity of cigar use). Analyses were repeated for each product, defined as brand by flavor for cigarettes vs brand alone for cigars (NSDUH lacks cigar flavor questions). To identify products with disproportionate underage use, each product’s consumption-weighted underage share (percentage of consumption due to consumers aged <21 years) was compared with the rest of their market’s underage share using Wald tests. Analyses used Stata, version 16.1 (StataCorp LLC), and followed the Strengthening the Reporting of Observational Studies in Epidemiology (STROBE) reporting guideline. The significance threshold was 2-tailed *P* = .05.

## Results

**Figure 1.**
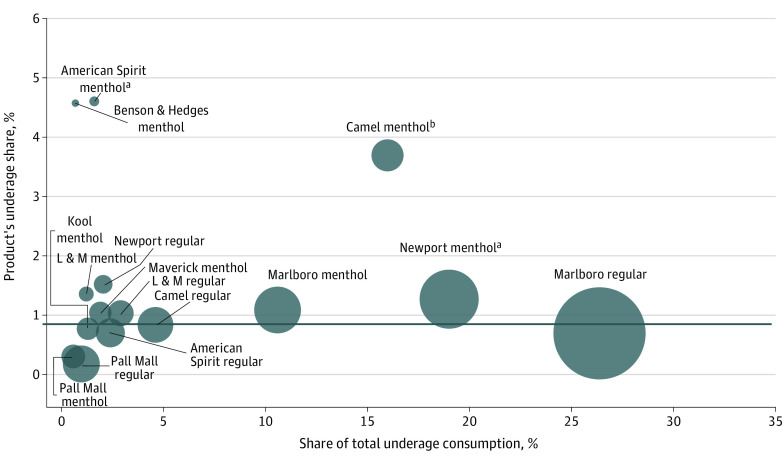
Proportion of Cigarette Consumption Due to Underage Use by Product The 15 products with the highest share of total underage (<21 years) consumption are shown, with each circle’s area reflecting the product’s total market share (calculated by dividing the product’s weighted consumption by the total weighted consumption for all cigarettes). Each product’s underage share was estimated by dividing the product’s weighted underage consumption by its total weighted consumption. The dark gray horizontal line represents the mean underage share of 0.8%. *P* values were derived from Wald tests comparing each product’s underage share with that of the rest of the cigarette market (ie, across all other cigarette products). ^a^*P* < .05. ^b^*P* < .01.

From January 1, 2020, to December 31, 2021, underage persons accounted for 0.8% of cigarettes smoked and 6.0% of cigar smoking days in the US. Three cigarette products had significantly larger underage shares than the rest of the market: American Spirit menthol (4.6%; *P* = .02), Camel menthol (3.7%; *P* < .001), and Newport menthol (1.3%; *P* = .04) (Figure 1). Together, they comprised 15.2% of total cigarette consumption but 36.6% of underage use. Notably, profitability did not require disproportionate underage use; nonmenthol Marlboro cigarettes, the market leader, covered 29.4% of overall use and 26.3% of underage consumption.

**Figure 2.**
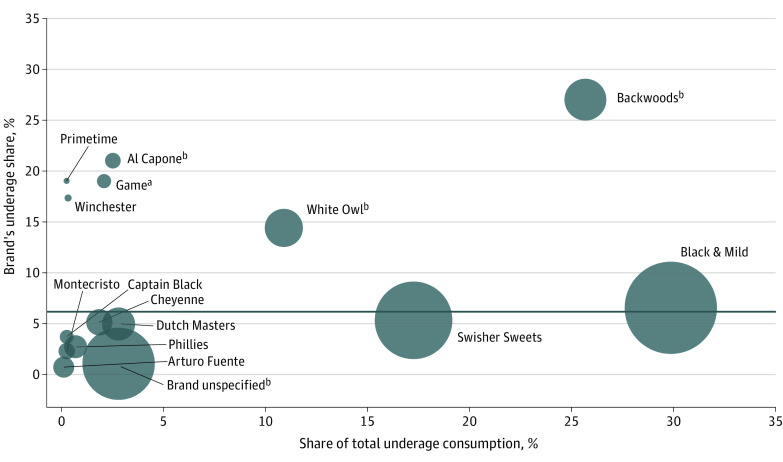
Proportion of Cigar Consumption Due to Underage Use by Brand The 15 brands with the highest share of total underage (<21 years) consumption are shown, with each circle’s area reflecting the brand’s total market share (calculated by dividing the weighted consumption days for each brand by the total weighted consumption days for all cigars). Each brand’s underage share was estimated by dividing the brand’s weighted consumption days by underage persons by its total weighted consumption days. The dark gray horizontal line represents the mean underage share of 6.0%. *P* values were derived from Wald tests comparing each product’s underage share with that of the rest of the cigar market (ie, across all other cigar brands). ^a^*P* < .05. ^b^*P* < .01.

Four cigar brands had significantly higher underage shares than the rest of the market: Backwoods (27.0%; *P* < .001), Al Capone (21.0%; *P* = .002), Game (19.0%; *P* = .01), and White Owl (14.4%; *P* < .001) (Figure 2). Together, they comprised 11.7% of total consumption but 41.5% of underage use. Yet competitors thrived without disproportionate youth use; Swisher Sweets and Black & Mild covered 47.2% of underage use and 46.9% of total consumption.

## Discussion

This survey study found that over one-third of underage cigarette and cigar use stemmed from a handful of brands with disproportionate underage use. Competitors’ success shows that disproportionate youth use is not required for profit.

These findings call for a targeted regulatory response; companies whose products exhibit disproportionate underage use should face substantive penalties. While proposed menthol and flavor prohibitions may help reduce youth use, they are insufficient alone. Consider California’s menthol ban: firms introduced cigarettes with nonmenthol coolants and promoted them to menthol smokers.^4^ Eliminating underage use requires making it costly to firms.

While this approach will provoke legal challenges from tobacco companies, it has been suggested previously. The June 1997 tobacco settlement’s lookback provisions^5^ specified time-dependent decreases in underage smoking, which, if unmet, would trigger a surcharge based on estimated lifetime profits from underage users. That policy was not implemented.

It is time for Congress to revisit such penalties by amending subsection 919(b) of the Food, Drug, and Cosmetic Act^6^ to assess greater user fees from firms with products exhibiting disproportionate underage consumption and extend user fees to newer nicotine products like e-cigarettes. Adding e-cigarette brand questions to the NSDUH would enable annual analyses of their underage shares by brand (alongside cigarettes and cigars) to inform contingent penalties as markets evolve. Study limitations include reliance on self-reported data subject to response biases. With so few products responsible for a disproportionate share of underage cigarette and cigar use, targeted penalties offer a means to further decrease youth use by realigning profit motives toward public health.

## Notes

Supplement.
Data Sharing Statement
