# Supplementary material for: Brand Differences in Underage Tobacco Use as Evidence for Targeted Sanctions—Reviving the Lookback
Source: JAMA Health Forum. 2023 Oct 6;4(10):e233463. doi: 10.1001/jamahealthforum.2023.3463 (PMC10559178; doi:10.1001/jamahealthforum.2023.3463)
Supplement: Supplement. — Data Sharing Statement [file jamahealthforum-e233463-s001.pdf]

Data Sharing Statement

Friedman. Brand Differences in Underage Tobacco Use as Evidence for Targeted Sanctions—Reviving the Lookback. *JAMA Health Forum*. Published October 06, 2023. doi:10.1001/jamahealthforum.2023.3463

Data
Data available: No

Additional Information
Explanation for why data not available: This paper uses publicly available data from the 2020-2021 National Survey on Drug Use and Health. While we are not permitted to disseminate these data as intermediaries, it can be obtained online.
